# The impact of dynamic caudal type homeobox 2 expression on the differentiation of human trophoblast lineage during implantation

**DOI:** 10.1111/cpr.13729

**Published:** 2024-08-19

**Authors:** Lujuan Rong, Lifeng Xiang, Zongyong Ai, Baohua Niu, Yaqing Wang, Yu Yin, Chun Feng, Gaohui Shi, Tingwei Chen, Jie Yang, Xi Luo, Yun Bai, Xiaoting Zhou, Xiaoping Liu, Haishan Zheng, Yang Ke, Tianqing Li, Ze Wu

**Affiliations:** ^1^ Faculty of Life Science and Technology Kunming University of Science and Technology Kunming Yunnan China; ^2^ Department of Reproductive Medicine The First People's Hospital of Yunnan Province Kunming Yunnan China; ^3^ The Affiliated Hospital of Kunming University of Science and Technology Kunming Yunnan China; ^4^ Department of Reproductive Medicine, NHC Key Laboratory of Healthy Birth and Birth Defect Prevention in Western China (Co‐building) The First People's Hospital of Yunnan Province Kunming Yunnan China; ^5^ State Key Laboratory of Primate Biomedical Research, Institute of Primate Translational Medicine Kunming University of Science and Technology Kunming Yunnan China; ^6^ KUST‐YPFPH Reproductive Medicine Joint Research Center Kunming Yunnan China; ^7^ University of Science and Technology of China Hefei Anhui China; ^8^ Suzhou Institute for Advanced Research University of Science and Technology of China Suzhou Jiangsu China

## Abstract

The trophoblast lineage differentiation represents a rate‐limiting step in successful embryo implantation. Adhesion, invasion and migration processes within the trophoblast are governed by several transcription factors. Among them, *CDX2* is a critical regulator shaping the destiny of the trophoblast. While its altered expression is a linchpin initiating embryo implantation in mice, the precise influence of *CDX2* on the functionality and lineage differentiation of early human trophoblast remains unclear. In this study, we employed well‐established human trophoblast stem cell (hTSC) lines with *CDX2* overexpression coupled with a 3D in vitro culture system for early human embryos. We revealed that the downregulation of *CDX2* is a prerequisite for syncytialization during human embryo implantation based on immunofluorescence, transcriptome analysis, CUT‐tag sequencing and the construction of 3D human trophoblast organoids. While *CDX2* overexpression inhibited syncytialization, it propelled hTSC proliferation and invasive migration. *CDX2* exerted its influence by interacting with *CGA*, *PTGS2*, *GCM1*, *LEF1* and *CDH2*, thereby hindering premature differentiation of the syncytiotrophoblast. *CDX2* overexpression enhanced the epithelial–mesenchymal transition of human trophoblast organoids. In summary, our study provides insights into the molecular characteristics of trophoblast differentiation and development in humans, laying a theoretical foundation for advancing research in embryo implantation.

## Graphical abstract

In the study, we found that downregulation of CDX2 during implantation is a prerequisite for human trophoblast cell fusion. In addition, sustained high expression of CDX2 in the human trophoblast leads to significant downregulation of fusion genes and promotes human trophoblast cell proliferation, as well as enhanced invasive migration.

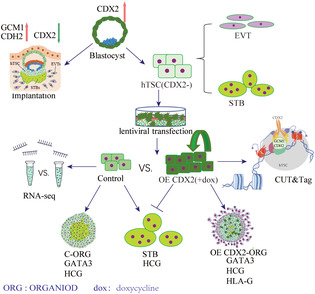

## 1 INTRODUCTION

Human beings exhibit lower fertility rates compared to other mammals. With the proportion of infertile individuals increasing each year, effectively enhancing fertility rates in the new era has become a pressing societal task.^1^, ^2^ Statistical data suggest that 60% of pregnancy losses occur during embryo implantation.^3^ The process wherein the blastocyst makes its initial contact with the endometrium, establishing a direct physiological relationship, is called embryo implantation. This process occurs from Day 6 to Day 8 after fertilisation, comprising three distinct stages: apposition, adhesion and invasion.^4^ The trophectoderm is the earliest cell type to come into contact with the endometrium. Beyond its role in secreting various cytokines to facilitate embryo apposition and adhesion to the endometrial epithelium, the trophectoderm undergoes lineage differentiation, giving rise to different cell lineages. These include cytotrophoblast with pluripotency, multinucleated syncytiotrophoblast and extravillous trophoblast.^5^, ^6^ Throughout embryo implantation, the differentiated trophoblast lineage of the blastocyst generates various cytokines affecting bidirectional communication between the embryo and the endometrium. Anomalies in this differentiation process often lead to implantation failure.^7^, ^8^

As a critical factor in embryo implantation, syncytiotrophoblast emerge early during the embryo implantation phase. It mainly includes two types: the ‘primary syncytiotrophoblast’ for invading the endometrium and the ‘secondary syncytiotrophoblast’ characterised by strong secretion functions.^9^ Syncytiotrophoblast functions as a nexus for nutritional exchange between the mother and foetus and acts as a physical barrier to protect the mother from pathogenic infections. It actively produces steroids, pregnancy hormones and immune‐regulatory factors, orchestrating a transformation of the maternal immune microenvironment to enhance maternal–foetal immune tolerance.^10^, ^11^ The cell fusion of syncytiotrophoblast is strictly regulated by molecular mechanisms.^12^, ^13^, ^14^ Notably, the primary syncytiotrophoblast exhibits an elevated expression of matrix metalloproteinases *MMP2* and *MMP9*, effectively reshaping the endometrial stroma and promoting embryonic invasion into the endometrial stroma.^15^, ^16^ Moreover, homologues of key transcription factors associated with trophectoderm development in mice, including *CDX2*, *TFAP2C*, *GCM1* and *GATA3*, have been identified in the human trophectoderm. Importantly, *GCA*, *GCM1*, *PSG* and *CGB* are unique markers for syncytiotrophoblast, exhibiting high expression levels. These genes are crucial in the formation and maintenance of syncytiotrophoblast. Disruptions in the function or formation of syncytiotrophoblast can directly impact foetal development and the mother's adaptability to pregnancy.^17^ Exploring the molecular mechanisms associated with syncytiotrophoblast development offers a fresh perspective for enhancing implantation and elevating pregnancy rates.

*CDX2* is a member of the caudal‐related homeobox transcription factor gene family. *Cdx2* is crucial transcriptional activator trophectoderm development in mice.^18^ Despite the high expression of *CDX2* in the trophectoderm of mice, non‐human primates and human blastocysts, its expression undergoes a marked spatiotemporal decrease with blastocyst implantation.^19^, ^20^ Overexpression of *Cdx2* can lead to the disturbance of trophoblast differentiation and adhesion at the implantation site in mice.^21^, ^22^ However, whether *CDX2* acts as a switch for trophoblast lineage differentiation during human embryo implantation remains unclear. Therefore, exploring the role of *CDX2* expression in human trophoblast lineage differentiation contributes to understanding the molecular characteristics and implantation potential of trophoblast differentiation during this stage, thereby providing a theoretical basis for a deeper understanding of embryo implantation and the formation of the placental barrier.

In this study, we posit that aberrant *CDX2* expression can alter the trophoblast's fate and functionality, influencing the implantation outcome. Based on previously established 3D culture systems of human embryos and other experimental approaches, we examined the functional role of *CDX2* overexpression in human trophoblast lineage differentiation and elucidated its underlying mode of action. The findings from this study contribute to the enhancement of our understanding of the molecular biology framework regarding the trophoblast cell‐fate transition during human embryo implantation.

## 2 MATERIALS AND METHODS

### 2.1 Ethics statement

This work was approved by the Medicine Ethics Committee of The First People's Hospital of Yunnan Province (2017LS[K]NO.035).^22^ All donated embryos in this study were surplus frozen embryos from couples who already had at least one healthy baby after in vitro fertilisation clinic treatment. The informed consent process for embryo donation complied with International Society for Stem Cell Research (ISSCR) ‘Guide lines for Stem Cell Research and Clinical Translation (2016)’ and ‘Ethical Guidelines for Human Embryonic Stem Cell Research (2003)’ jointly issued by the Ministry of Science and Technology and the Ministry of Health of People's Republic of China. The Medicine Ethics Committee of The First People's Hospital of Yunnan Province is composed of nine members, including lawyers, scientists and clinicians with relevant expertise. The Committee evaluated the scientific merit and ethical justification of this study and conducted a full review of the donations and use of these samples. All donor couples signed informed consents for voluntary donations of surplus embryos for human embryo development study at the Department of Reproductive Medicine in the First People's Hospital of Yunnan Province. No financial inducements were offered for the donations. In the process, couples were informed that their embryos would be used to study the developmental mechanisms of human embryos and that their donation would not affect their in vitro fertilisation cycle. The culture of all embryos was terminated at Day 8 after fertilisation or upon the appearance of primitive streak anlage.

### 2.2 Human embryo and trophoblast culture

In vitro delayed culture of early human embryos was performed using a 3D culture system previously published by our research group.^22^ Human trophoblast cells were seeded into 6‐well plates coated with 5 μg/mL type IV collagen and incubated at 37°C for at least 1 h. The cell density in each well was 0.5–1 × 10^6^, and 2 mL of medium containing Dulbecco's modified eagle medium (DMEM)/F12(gibco 11330‐032) as the base, supplemented with 0.1 mM mercaptoethanol (Thermo Fisher Scientific Cat# 21985023), 0.2% fetal bovine serum (FBS) (Thermo Fisher Scientific Cat# 16141‐079), 0.3% bovine serum albumin (BSA) (Wako Cat# 017‐22231), 1% insulin‐transferrin‐selenium‐ethanolamine media supplement (ITS‐X) supplement (Wako Cat# 094‐06761), 1.5 μg/mL L‐ascorbic acid (Wako Cat# 013‐12061), 50 ng/mL epidermal growth factor (EGF) (Wako Cat# 053‐07871), 2 μM CHIR99021 (Wako Cat# 038‐23101), 0.5 μM A83‐01 (Wako Cat# 035‐24113), 1 μM SB431542 (Wako Cat# 031‐24291), 0.8 mM valproic acid (VPA) (Wako Cat# 227‐01071) and 5 μM Y27632 (Wako Cat# 257‐00511)was added to each well. The cells were then cultured at 37°C with 5% CO_2_, with medium changes every 2 days. When the cell confluence reached 70%–80%, Tryple (Thermo Fisher Scientific Cat# 12604021) solution was added, and cells were digested at 37°C for 5–7 min. Subculturing was performed at a ratio of 1:2–1:4 in new 6‐well plates coated with 5 μg/mL type IV collagen (Sigma‐Aldrich C7521) and incubated for at least 1 h at 37°C. For cell cryopreservation, cells were resuspended in a freezing solution composed of 45% culture medium, 45% FBS and 10% Dimethyl Sulfoxide (Sigma‐Aldrich C7521).

### 2.3 Immunofluorescence staining

Cells were fixed with 4% paraformaldehyde (PFA), permeabilised, and blocked with PBS containing 0.5% Triton‐X and 3% BSA. The primary antibody was then added in 1% BSA at the appropriate dilution and quantity, followed by overnight incubation at 4°C. After three washes with PBS containing 0.5% Tween‐20, the secondary antibody, prepared at the specified dilution and quantity, was added. Cells were incubated at room temperature for 2 h, followed by three additional washes with PBS containing 0.5% Tween‐20. Finally, 15% glycerol was added before imaging using a confocal microscope (Leica).

**Table:** 

Antibodies	Brand	Catalogue	Working dilution
Rabbit anti‐CDX2	Abcam	ab76541	1:500
Mouse anti‐GATA3	Thermo Fisher	MA1‐028	1:400
Rabbit anti‐TEAD4	Sigma	HPA056896	1:400
Mouse anti‐TP63	Abcam	ab735	1:500
Mouse anti‐TFAP2C	Santa Cruz	sc‐12,762	1:500
Mouse anti‐OCT4	Santa Cruz	SC‐5279	1:500
Goat anti‐NANOG	R&D Systems	AF19997	1:500
Rabbit anti‐CK7	Abcam	ab181598	1:500
Mouse anti‐E‐cadherin	Abcam	ab76055	1:500
Rabbit anti‐N‐cadherin	GeneTex	GTX1217345	1:500
Mouse anti‐HLA‐G	Abcam	ab52455	1:500
Mouse anti‐HCG	Abcam	ab9582	1:400
Rabbit anti‐Ki67	Cell Signalling	Cat.# 9129S	1:500
Mouse anti‐GCM1	Santa cruz	sc‐101,173	1:500
Rabbit anti‐β‐catenin	Cell Signalling	Cat.# 8480S	1:500
Donkey anti‐mouse Alexa Fluor 568	Thermo Fisher	A10037	1:500
donkey anti‐rabbit Alexa Fluor 644	Thermo Fisher	A31573	1:500
donkey anti‐goat Alexa Fluor 488	Thermo Fisher	A11055	1:500
DAPI solution (ready‐to use)	Solarbio	C0065	1:800

### 2.4 Construction of *CDX2* recombinant plasmid

The human *CDX2* gene sequence was referenced from the national center for biotechnology information database, and *CDX2* primers were designed using Snapgene based on this sequence. The amplification of *CDX2* was performed using RNA from human embryonic stem cells with the designed primers. The primer sequence is CDX22‐*EcoRI*‐F: CGgaattcCGATGTACGTGAGCTACCTCCTGGAC CDX2‐*BamHI*‐R: CGggatccCGTCACTGGGTGACGGTGGGGTTTAG. After confirming the correctness of the *CDX2* sequence through sequencing, a *CDX2‐pLVX‐TetOne‐EGFP* recombinant plasmid was constructed. Successful construction of the recombinant plasmid was confirmed through double enzyme digestion and bacterial liquid sequencing.

### 2.5 Transfection of human trophoblast cells with lentivirus

Following the manufacturer's instructions, we utilised a calcium phosphate transfection kit to generate DNA calcium phosphate precipitates, facilitating the adhesion of *CDX2* DNA to the cell surface and subsequent capture via endocytosis in 293T cells. After 48 h of transfection in 293T cells, the supernatant was harvested, purified and concentrated using an ultrafiltration column to obtain the lentivirus, with subsequent determination of the viral titre. Prior to transfection, human trophoblast stem cell (hTSC) cells were seeded at a density of 1 × 10^4^ cells per well in a 24‐well plate. After 48 h of cultivation at 37°C, the medium was replaced with a fresh hTSC culture medium containing 20 μL of virus. Following an 8‐h incubation at 37°C, the medium was again replaced with a fresh hTSC culture medium, and the cells were further cultured for 48 h. The proportion of green fluorescent protein (GFP)+ cells was observed using a laser confocal microscope (Leica TCS SP8, Germany). GFP+ trophoblast cells were sorted using flow cytometry. In this study, we used the same method to transfect three different strains of trophoblast stem cells.

### 2.6 GFP‐positive cells obtained through fluorescence‐activated cell sorting (FACS)

After treating transfected cells with Tryple at 37°C for 6 min to digest them into single cells, the cells were collected into a centrifuge tube, centrifuged at 1000 rpm for 5 min, and the supernatant was discarded. The cells were resuspended in 1 mL of PBS, and the entire suspension was transferred for fluorescence‐activated cell sorting (FACS) to isolate human trophoblast cells expressing GFP.

### 2.7 In vitro directed differentiation into syncytiotrophoblast and EVTs

When the confluence of human trophoblast cells reached 80%, the cells were subjected to digestion at 37°C for 10–15 min. Cells of 1.875 × 10^4^ were seeded into four‐well plates precoated with 1 μg/mL type IV collagen and incubated for at least 1 h to induce the differentiation of trophoblast cells into EVTs. Subsequently, 500 μL of Extravillous trophoblast (EVT) (3D) culture medium was added, consisting of DMEM/F12, 0.1 mM mercaptoethanol, 0.3% BSA, 1% ITS‐X supplement, 100 ng/mL NRG1(Cell Signalling Cat# 5218SC), 7.5 μM A83‐01, 2.5 μM Y27632, and 4% Knock Out Serum Replacement (Thermo Fisher Scientific Cat# 10828028). The cells were cultured using this medium. Simultaneously, during cell resuspension, 2% Matrigel (final concentration) was added to the culture medium. After 3 days, an EVT culture medium without NRG1 and with a final Matrigel (Corning Cat# 354234) concentration of 0.5% was applied. By the sixth day, the cell confluence reached approximately 80%. At this point, Tryple solution was utilised for digestion at 37°C for 5–7 min. The cells were passaged at a 1:2 ratio into a new four‐well plate precoated with 1 μg/mL type IV collagen and incubated for at least 1 h before further cultivation. Subsequently, the cells were cultured for an additional 2 days in EVT culture medium (no NRG1 and KnockOut™ Serum Replacement) with a final Matrigel concentration of 0.5% before proceeding to immunofluorescence staining and imaging using a Leica confocal microscope.

Cells of 6.25 × 10^4^ were seeded into a 24‐well plate precoated with 2.5 μg/mL type IV collagen and incubated for at least 1 h to induce the differentiation of trophoblast cells into syncytiotrophoblasts (3D). Then, 750 μL of syncytiotrophoblast (3D) culture medium was added, composed of DMEM/F12, 0.1 mM mercaptoethanol, 0.3% BSA, 1% ITS‐X supplement, 2.5 μM Y27632, 50 ng/mL EGF, 2 μM Foskolin (Wako Cat# 067‐02191), and 4% KOSR. After 3 days, an equal volume of fresh culture medium was added for another 3 days of cultivation. On the sixth day, the cells were fixed, and immunofluorescence staining was performed, followed by imaging using a Leica confocal microscope.

### 2.8 Construction of 3D trophoblast organoids

The human trophoblast organoids were established according to a previously published 3D culture system [34]. *CDX2*‐overexpressing trophoblast cells were digested into single cells. In a culture medium of trophoblast organoid containing 40 μL of 60% Matrigel, 6 × 10^4^ cells were added, thoroughly mixed, and seeded into the central well of a 24‐well plate. Initially, the seeded plate was placed in a 37°C, 5% CO_2_ incubator in an upright position for 2 min. Subsequently, the plate was inverted for 15 min under the same incubation conditions to ensure uniform cell distribution within the Matrigel droplet. Afterward, the plate was retrieved, and 500 μL of trophoblast organoid culture medium was added to each well along the plate edges. The cells were cultured at 37°C, 5% CO_2_. Once the trophoblast organoid diameter reached 200–400 μm, the organoids were collected, fixed with 4% PFA for 40 min, dehydrated in 20% sucrose for 2–3 min, embedded in optimal cutting temperature compound for frozen sectioning, and analysed the lineage composition of trophoblast organoids using immunofluorescence.

### 2.9 Invasion and migration assays

A 24‐well plate with 8 μm pore size inserts of 6.5 mm diameter (Corning 14322032) was employed to assess the invasive capabilities of trophoblast cells. For the invasion assay, 200 μL of FBS‐free culture medium was added to transfected GFP‐positive human trophoblast cells and *CDX2*‐overexpressing cells (cell density: 4 × 10^4^/well). Matrigel (80 μL; dilution 1:4; Sigma, St. Louis, MO) was applied to the upper chamber, while the lower chamber received medium containing 10% FBS (500 μL). The seeded plates were incubated at 37°C for 2 days. Following formaldehyde fixation, the cells were stained with 0.1% crystal violet staining solution (Biosharp BL802A). Subsequently, the number of crystal violet‐labelled cells was recorded under an optical microscope at 100× magnification in different fields of view. For the migration assay, GFP‐positive human trophoblast cells and *CDX2*‐overexpressing cells (cell density: 5 × 10^4^/well) were seeded in 24‐well plates coated with 5 μg/mL type IV collagen at 37°C for at least 1 h. The cells were cultured in hTSC medium containing 10% FBS at 37°C with 5% CO_2_ for 24 h. After complete cell adhesion, a line was scratched along the centre of each well, and the wells were washed three times with PBS to remove the remaining cells. Subsequently, 500 μL of culture medium without FBS was added to each well for cultivation. The growth status of cells at 0, 24 and 48 h was observed and recorded using an optical microscope.

### 2.10 Quantitative Polymerase Chain Reaction (q‐PCR)

Total RNA from directed trophoblast differentiation under various experimental conditions was extracted using TRIzol reagent. Subsequently, a microRNA reverse transcription kit was used, with U6 as the normalisation reference. mRNA detection was performed using an mRNA reverse transcription kit. SYBR Green quantitative PCR analysis was conducted on the 7500 Real‐Time PCR system. The relative gene expression levels were calculated using the −ΔΔ2Ct method. The primer sequences used in these experiments were as follows: human CGA‐F (forward): CACTCCACTAAGGTCCAAGAAGA, human CGA‐R (reverse): CCGTGTGGTTCTCCACTTTGA; human PSG3‐F (forward): TCGTAAAGCGAGGTGATGGG; human PSG3‐R (reverse): AAGCTCACAGCCTCCATGTC; human CGB‐F (forward): ACCCTGGCTGTGGAGAAGGAG, human CGB‐R (reverse): ATGGACTCGAAGCGCACATCG.

### 2.11 Generation and sequencing of CUT‐tag libraries

CUT‐tag treatment was performed using the hyperactive in situ ChIP Library Prep Kit (Vazyme Biotech, TD903) following the manufacturer's instructions. After phenol‐chloroform extraction and ethanol precipitation, the libraries underwent PCR amplification. All libraries were subjected to sequencing using the Illumina NovaSeq 6000 platform.

### 2.12 Transcriptome sequencing analysis

After culturing trophoblast cells on a plate with stem cell and differentiation culture medium for 3 days, both stem cells and differentiated trophoblast cells were collected. Total RNA was isolated from the samples using Trizol reagent. Standard RNA sequencing libraries were prepared using the Ribooff rRNA Depletion Kit (Illumina) and KC‐Digital™ Standard mRNA Library Prep Kit for Illumina (Seqhealth) according to the manufacturer's instructions. Libraries with products corresponding to 200–500 bps were enriched, quantified, and sequenced on the NovaSeq 6000 sequencer (Illumina) with a PE150 sequencing model. Sequencing data were analysed using the standard RNA‐seq protocol. Read mapping was performed using the STAR software (version 2.5.0) to map reads to the Homo sapiens reference genome (GRCh38). STAR software (version 2.5.3a) with default parameters was employed to map reads to the *Homo sapiens* reference genome from Homoserines (GRCh38).

### 2.13 Data analysis

Statistical analysis was conducted using GraphPad Prism 9.0. Unless otherwise specified, most experimental data underwent a two‐tailed Student's *t*‐test or analysis of variance, with a significance threshold set at *p* < 0.05. The results are reported as the mean ± SE based on at least three independent experiments.

## 3 RESULTS

### 3.1 *CDX2* as a prerequisite for the syncytialization during implantation

**FIGURE 1:**
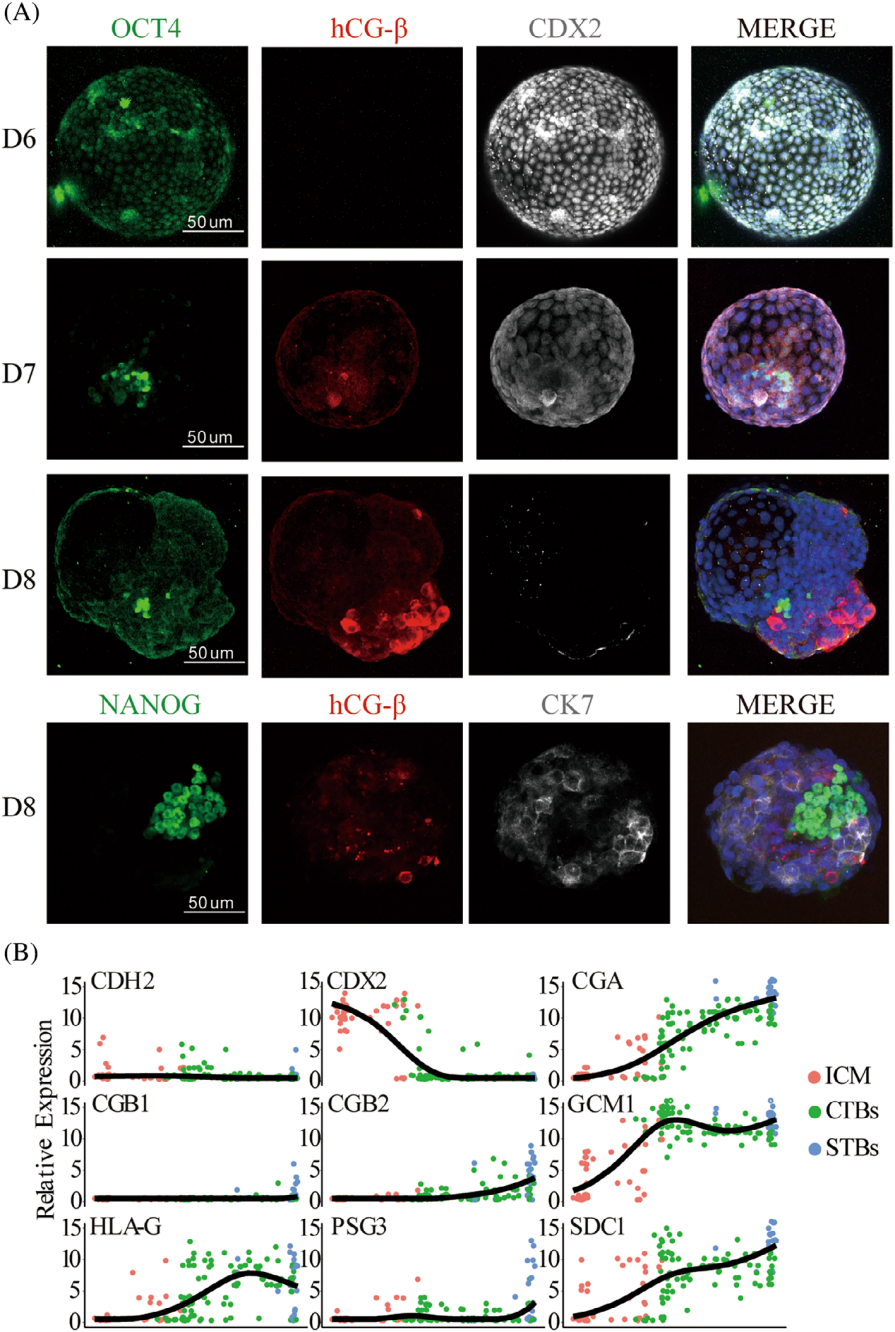
Expression dynamics of *CDX2* during early human embryo implantation and its correlation with syncytialization. (A) Immunofluorescence assessment of *CDX2* expression and syncytiotrophoblast differentiation in human embryos during implantation. *OCT4* and *NANOG* were used for epiblasts characterisation, *CDX2* was used for trophectoderm characterisation, *hCG‐β* was used for syncytiotrophoblast characterisation, *CK7* was used for trophoblast stem cell characterisation. Scale bars are 50 μm.(B) Dot plots showing the expression of *CDX2*, *GCM1*, *CGA*, *CGB1*, *CGB2*, *PSG3*, *SDC1*, *CDH2* and *HLA‐G* in human embryonic‐derived inner cell mass (ICMs), cytotrophoblasts (CTBs) and syncytiotrophoblasts (STBs) from d.f. 6 to d.f. 9. *GCM1*, *CGA*, *CGB1*, *CGB2* and *PSG3* are markers for syncytiotrophoblast, *CDH2* and *HLA‐G* are associated with invasive migration.

The understanding remains elusive for *CDX2*'s expression changes and functions during human embryo implantation due to substantial species variations and conflicting outcomes.^23^, ^24^ To explore the dynamics of *CDX2* expression during human embryo implantation and its impact on trophectoderm lineage differentiation, we employed the established 3D culture system to conduct in vitro delayed cultivation of discarded embryos.^22^ Subsequently, embryos from Day 6 to Day 8 were collected to explore the link between *CDX2* expression and trophoblast lineage differentiation at the protein and gene level. The results of the immunofluorescence assay showed that during implantation, a notable reduction in *CDX2* expression in the trophectoderm coincided with the initiation of trophoblast differentiation, resulting in the formation of syncytiotrophoblast expressing *hCG‐ß* (Figure 1A). We hypothesised that downregulating *CDX2* could create conditions conducive to the expression of key genes involved in syncytiotrophoblast fate determination, such as *GCM1*, *CGA*, *CGB1*, *CGB2*, *PSG3* and *SDC1*. To test this hypothesis, we compared the expression of *CDX2*, these key genes and *CDH2* and *HLA‐G*, in the inner cell mass, cytotrophoblasts and syncytiotrophoblasts during embryo implantation. The results confirmed downregulated *CDX2* expression, but *CGA*, *GCM1* and *SDC1* exhibited substantial upregulation. In addition, *CGB1*, *CGB2* and *PSG3* also show upward trends (Figure 1B). *CGB1* and *CGB2* demonstrated a close association with *hCG‐ß* synthesis and secretion in the syncytiotrophoblast. These results emphasise the crucial role of downregulated *CDX2* expression in syncytialization during implantation.

### 3.2 The model of *CDX2* overexpression for early hTSCs

**FIGURE 2:**
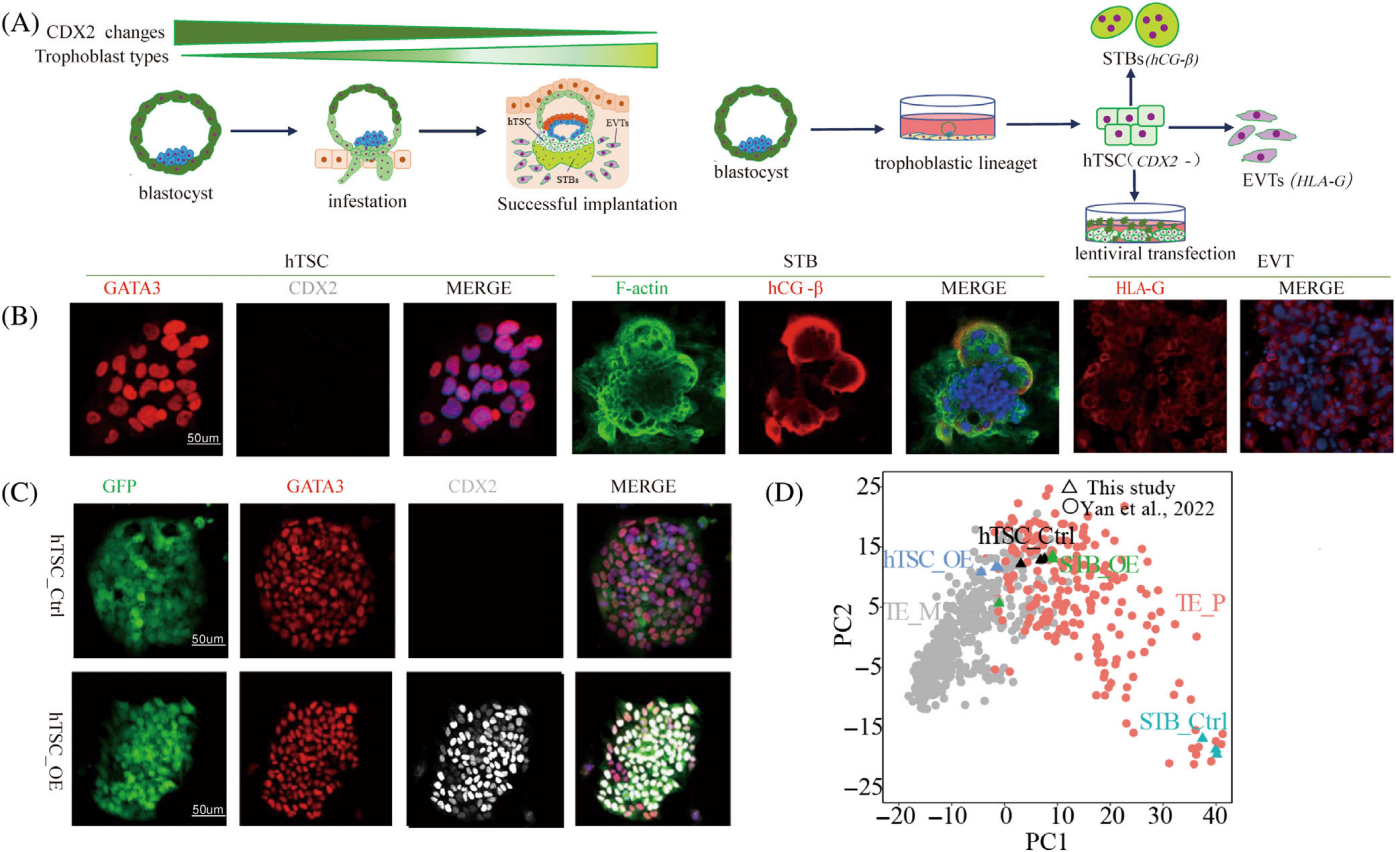
Early human trophoblast stem cell (hTSCs) model with *CDX2* overexpression. (A) Correlation between *CDX2* expression during human embryo implantation and trophoblast lineage differentiation, and the embryonic‐derived hTSC lines established; (B) immunofluorescence of cell types and differentiation lineages derived from cell lines of the blastocyst. EVT, extravillous trophoblast; STB, syncytiotrophoblast. *GATA3* and *CDX2* were used for hTSC characterisation, *F‐actin* and *hCG‐β* were used for STB characterisation, *HLA‐G* was used for EVT characterisation, staining. Scale bars are 50 μm; (C) FACS‐purified hTSCs that have been successfully transfection, with immunofluorescence staining to identify their purity, scale bars are 50 μm. hTSC_Ctrl: trophoblast stem cells carrying GFP fluorescence without exogenous addition of doxycycline induction，hTSC_OE: addition of doxycycline induces *CDX2* overexpression in trophoblast stem cells; (D) principal component analysis showing the effect of *CDX2* overexpression on the similarity between hTSCs or syncytiotrophoblasts and human polar and mural trophectoderm, using all expressed genes as input. Please note that hTSC_OE clusters with TE_M, hTSC_Ctrl, STB_Ctrl and STB_OE polar trophectoderm cluster together, but STB_OE is close to hTSC_Ctrl, and *CDX2* overexpression hinders syncytiotrophoblasts differentiation. STB_Ctrl, hTSC_Ctrl differentiates to form syncytiotrophoblasts; STB_OE, hTSC_OE differentiates to form syncytiotrophoblasts; TE_M, mural trophectoderm; TE_P, polar trophectoderm.

The diversity of trophoblast lineages during implantation, ethical and material constraints hinder the exploration of *CDX2* functionality at the level of the human embryo. In addition, naive and primed embryonic stem cells, although induced exogenously to form trophoblast‐like cells, only transiently express *CDX2*.^25^, ^26^, ^27^ To overcome this issue, we conducted morphological assessments of in vitro cultured embryos using optical microscopy and selected suitable blastocysts. Subsequently, following the methodology reported by Hiroaki Okae, we established hTSC lines derived from human blastocysts, the established cell lines were identified by the recognised standard of trophoblast identity, and it was clear that they could be used as a cell model for the study of early human trophoblast development.^28^, ^29^, ^30^ Employing directed differentiation for syncytiotrophoblast and EVTs, we cultured these cells in vitro (Figure 2A). Immunofluorescence identification confirmed the multi‐lineage differentiation capability of these cells as hTSCs; however, they did not express *CDX2* (Figure 2B). To obtain a trophoblast model expressing *CDX2*, we introduced *CDX2* overexpression through lentiviral transfection into the hTSCs. Following FACS purification and immunofluorescence results indicated that after transfection, the cells expressed GFP and *CDX2* in the same site upon doxycycline induction. GFP served as a fluorescence label to assess the success of transfection, we successfully obtained the early hTSC lines with *CDX2* overexpression (Figure 2C). The trophectoderm of the mammalian blastocyst can be divided into polar trophoblast and mural trophoblast. During the implantation of the mouse embryo, the mural trophectoderm first comes into contact with the uterine endometrium, followed by downregulation of *Cdx2* expression and differentiation into placental giant cells. The polar trophectoderm retains high expression of *Cdx2* and heterogeneity in the expressions of *CDX2* and *GATA3* in the trophectoderm of the adherent and non‐implanted ends of the embryo has been confirmed by imaging in a monolayer co‐culture system of human embryos and endometrial epithelial cell lines.^16^, ^20^, ^21^ However, whether *CDX2* exists and is present in human mural and polar trophectoderm is still remains unknown.^23^, ^24^ We collected control and overexpressing *CDX2* hTSCs and the respective induced differentiation of syncytiotrophoblast for RNA‐seq analysis to clarify the lineage type of overexpressed *CDX2* in trophoblast cells. Subsequently, we combined these data with published scRNA‐seq data of blastocyst trophectoderm published by Yan Liying/Qiao Jie et al. for principal component analysis (PCA).^31^ Our results indicated that trophoblast stem cells overexpressing *CDX2* were more similar to mural trophectoderm, Although both control and differentiated syncytiotrophoblast cells were close to the polar trophectoderm, the syncytiotrophoblast derived from overexpressing *CDX2* was similar to the control trophoblast stem cells (Figure 2D).

In summary, we have observed that, consistent with implantation in mice, the downregulation of *CDX2* is a signalling nexus for initiating the trophectoderm lineage differentiation during implantation. Despite intrinsic defects in endogenous *CDX2* expression in hTSC derived from cell lines of the blastocyst, inducing exogenous *CDX2* expression does not impact the expression of pluripotency markers in these cells. *CDX2* overexpressing trophoblasts were closer to mural trophectoderm, reflecting that *CDX2* is also differentially expressed between human polar and mural trophectoderm. This cell model creates the conditions necessary for deciphering the role of *CDX2* in the differentiation of early human trophoblast lineage differentiation.

### 3.3 *CDX2* overexpression promotes proliferation of early hTSCs

**FIGURE 3:**
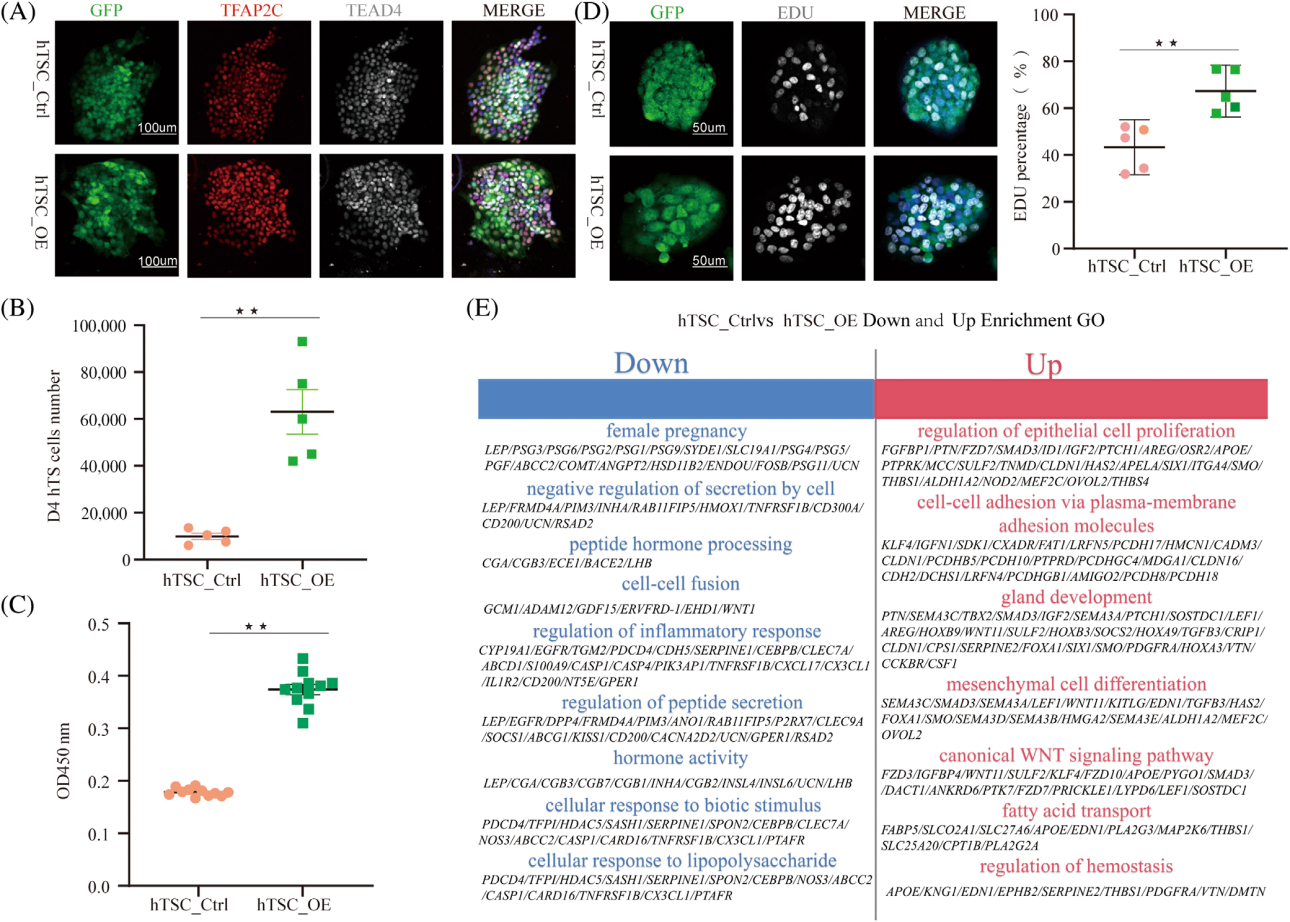
Impact of *CDX2* overexpression on proliferation of early human trophoblast stem cell (hTSCs). (A) Immunofluorescence images of hTSC markers; scale bars are 100 μm; (B) cell counting of hTSC_Ctrl and hTSC_OE. Grown in hTSC culture medium for 4 days, Data are presented as mean ± SE (*n* = 5 number of experimental replications), **p ≤ 0.01; (C) proliferative capacity assessed by CCK‐8 in hTSC_Ctrl and hTSC_OE, data are presented as mean ± SE (*n* = 11 number of replicates), **p ≤ 0.01; (D) fluorescence images of EDU‐labelled cells in hTSC_Ctrl and hTSC_OE groups, along with the statistical analysis, data are presented as mean ± SE (*n* = 5 number of experimental replications), **p ≤ 0.01; (E) GO enrichment analysis of significantly upregulated and downregulated genes in hTSC_Ctrl with hTSC_OE.

*Cdx2* is crucial in maintaining the fate and proliferation of trophoblast stem cells in mice, whereas in human trophectoderm, mural trophectoderm show more proliferative activity than polar trophectoderm.^23^, ^31^ Based on these findings, we investigate the impact of *CDX2* overexpression on the proliferative capacity of hTSCs at the cellular level, as well as to further clarify the conclusion that *CDX2* overexpressing hTSCs are mural trophectoderm. Different methods were employed for validation. Firstly, immunofluorescence identification showed that *CDX2* overexpression did not affect the pluripotency of hTSCs (Figure 3A). Subsequently, confirmation was obtained through cell counting, 5‐Ethynyl‐2'‐deoxyuridine (EDU) labelling, and CCK‐8 assay for proliferation, revealing that the *CDX2* expression significantly enhances the proliferative capacity of hTSCs (Figure 3B–D). Furthermore, RNA‐seq enrichment Gene Ontology (GO) analysis revealed that *CDX2* overexpression also promotes hTSCs proliferation and activates the essential fatty acid transport for membrane synthesis, along with the Wingless/Integrated (WNT) signalling pathway (Figure 3E). These results suggest that *CDX2* overexpression promotes the proliferation of early human trophoblast cells and enhances their cellular activity.

### 3.4 *CDX2* overexpression inhibits syncytialization

**FIGURE 4:**
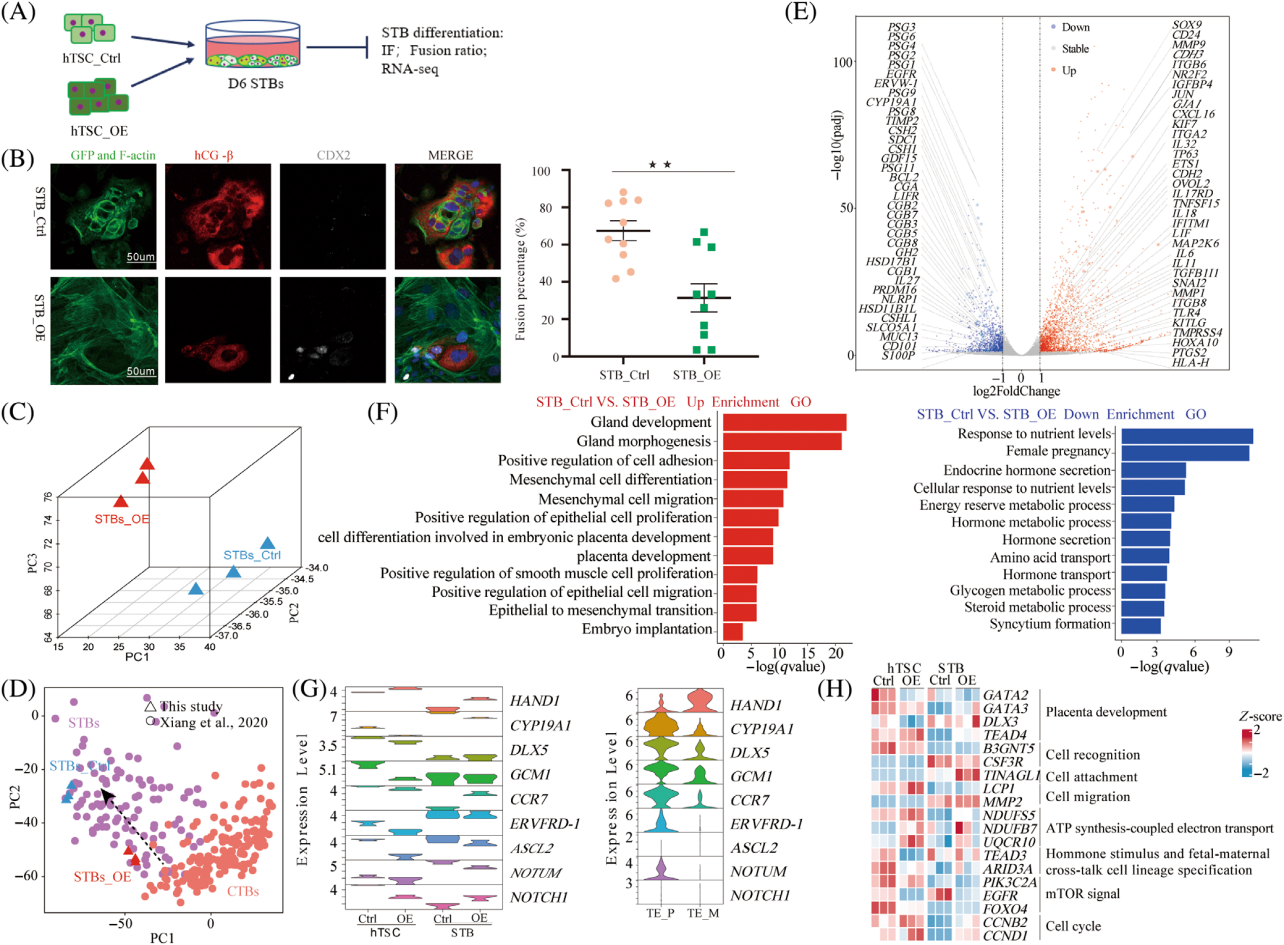
Inhibition of syncytialization by *CDX2* overexpression. (A) Directed differentiation into syncytiotrophoblast cells for the hTSC_Ctrl and hTSC_OE group. (B) Immunofluorescence images of syncytiotrophoblast markers on the sixth day of directed differentiation for the STB_Ctrl and STB_OE group. Statistical analysis of syncytiotrophoblast fusion ratio under directed differentiation. Syncytia were defined as cells with at least three nuclei. The calculation involved counting the number of DAPI‐stained nuclei and syncytia. The fusion ratio was calculated using the formula: ([*N* – *S*]/*T*) × 100, where *N* is the number of syncytial nuclei, *S* is the number of syncytia, and *T* is the total number of counted nuclei. Data are presented as mean ± SE (*n* = 10 number of experimental replications), **p ≤ 0.01. (C) The transcriptome on the sixth day of directed syncytiotrophoblast differentiation for the STB_Ctrl and STB_OE group. (D) Principal component analysis (PCA) plot after integrating the transcriptome group data of the STB_Ctrl and STB_OE with the cytotrophoblasts and syncytiotrophoblasts of human embryos from Day 6 to Day 14. The black dashed arrow indicates the differentiation trend. CTB, cytotrophoblast; STB, syncytiotrophoblast. (E) DGE analysis after 6 days of differentiation culture for the control group and OE CDX2 group. (F) Functional enrichment analysis of significantly upregulated or downregulated gene sets (false discovery rate <0.05). (G) Violin plot displaying the polar and mural trophectoderm related marker genes, and invasive migration‐related marker genes for hTSC_Ctrl and hTSC_OE, STB_Ctrl and STB_OE. (H) Expression patterns and functional annotation of common differentially expressed gene in stem cells and syncytiotrophoblast regarding implantation and non‐implantation poles in human and mouse embryos under *CDX2* overexpression conditions.

Syncytiotrophoblasts first originate from polar trophectoderm differentiation and are subsequently formed by the fusion of cytotrophoblast throughout pregnancy. The early syncytiotrophoblast promotes maternal–foetal interactions.^9^ Based on the previous studies that report the in vitro culture model expanding hTSCs, we observed that cells in the control group exhibited a few vacuole‐like structures, whereas cells with *CDX2* overexpression did not display such structures (Figure S1A). We speculate that these are syncytiotrophoblasts formed by spontaneous differentiation of hTSCs in culture. Subsequently, immunofluorescence staining and statistical analysis were employed to identify the cell type numbers with vacuole‐like structures. The results confirmed that overexpression of *CDX2* inhibits the spontaneous differentiation of *hTSCs* and reduces the formation of vacuole‐like syncytiotrophoblasts. (Figure S1B,C). Additionally, referring to reports related to trophoblast lineage differentiation, we selected classical markers (*CGB1*, *GCM1*, *PGF*, *MAFK*, *TBX3*, *ZNF292*, *ASCL2*, *PSG3*, *HES1*, *PSG6* and *TCL6*) that determine syncytialization. Transcriptome sequencing analysis revealed that these genes were downregulated in *CDX2* overexpressing hTSCs (Figure S1D). To elucidate the role of *CDX2* in syncytialization, we induced the differentiation of the cell model into syncytiotrophoblasts using an in vitro culture system and evaluated the differentiated cells through different methods (Figure 4A). Statistical results of immunofluorescence and fusion both indicate that *CDX2* overexpression can inhibit syncytialization of the hTSCs (Figure 4B).

To clarify the reasons for the inhibitory effect of *CDX2* overexpression on syncytialization, we designed different experiment groups and culture conditions (Figure S2A) for the directed differentiation of syncytiotrophoblast. Immunofluorescence was carried out to identify syncytialization under these experimental conditions. The inhibitory effect of *CDX2* on syncytiotrophoblasts depends on its expression (Figure S2B). This result is consistent with the results from the in vitro delayed culture of early human embryos (Figure 1A,B). The statistical results of the fusion ratio and q‐PCR analysis indicated that the capability of hTSC to respond to external differentiation signals was weakened after inducing *CDX2* overexpression (Figure S2C). Meanwhile, we observed during the differentiation culture that, compared to the control group, cells with *CDX2* overexpression still maintained a morphology similar to that of their stem cells (Figure S2D). Additionally, we used immunofluorescence to verify the expression of hTSC markers *TEAD4*, *TP63* and *CDX2* in this cell population. The results suggested that *CDX2* overexpression delayed the fate transition of hTSC towards syncytiotrophoblast differentiation (Figure S2E).

We subjected the RNA‐seq data of syncytiotrophoblast to correspondence analysis (CoA) for better comprehension of the effect of *CDX2* overexpression on syncytialization. The results revealed significant transcriptional differences between the cells undergoing *CDX2* overexpression‐induced differentiation and those in the control group (Figure 4C). After integrating this transcriptome data with previously published single‐cell data of human embryonic trophoblast stem cells and syncytiotrophoblast from Day 6 to Day 14, PCA demonstrated a distinct delay in the differentiation of trophoblasts into syncytiotrophoblast under *CDX2* overexpression (Figure 4D). Differential gene analysis suggested that in *CDX2* overexpressing differentiated cells, 2219 genes were upregulated, and 1231 genes were downregulated. Importantly, among these differentially expressed genes, those specific to syncytiotrophoblasts, such as the *PSG* family, *CGB* family and *SDC1*, showed significant downregulation. Simultaneously, genes associated with adhesion, cell cycle, invasion, migration and differentiation exhibited significant upregulation, aligning with the observed phenomena of heightened cellular proliferation, as well as impaired fusion and differentiation resulting from *CDX2* overexpression (Figure 4E). Upregulated genes included those linked to cell adhesion and migration (*MMP9*, *CD24* and *CDH2*) and placental development and differentiation (*NR2F2* and *PTGS2*). Downregulated genes were associated with nutrient exchange, hormone synthesis and secretion, and syncytialization (Figure 4F). This indicates that while *CDX2* overexpression compromises the structure and secretory function of the syncytiotrophoblast, it paradoxically addresses their deficiency in invasive migration.

Previous reports suggest that the polar trophectoderm is preferentially differentiated and highly expresses genes involved in cellular responses to hormonal stimuli and vascular development during the preparation for placental development. Simultaneously, cell cycle activity and energy metabolism were lower in the polar trophectoderm than in the mural trophectoderm.^31^ *CDX2* overexpressing trophoblast stem cells were more similar to the mural trophectoderm (Figure 2D), so we speculated that there was heterogeneity in *CDX2* expression in the trophectoderm of the human blastocyst, with preferential downregulation in the polar trophectoderm with the onset of implantation. To test this hypothesis, first, we selected genes reported to be important in trophoblast development, including *HAND1*, *CYP19A1*, *GCM1*, *ERVFRD‐1*, *DLX5*, *CCR7*, *NOTUM*, *ASCL2* and *NOTCH1*.^31^ We analysed the expression of these genes in hTSCs, syncytiotrophoblasts, polar trophectoderm and mural trophectoderm. A violin plot showed that *HAND1* was highly expressed in the mural trophectoderm, *CDX2* overexpressing hTSCs and syncytiotrophoblasts, whereas the expressions of *CYP19A1*, *DLX5*, *GCM1* and *NOTUM* were downregulated. Although the expressions of *DLX5* and *CCR7* in hTSCs were lower than that in polar trophectoderm, their expression was high in syncytiotrophoblasts originating from *CDX2* overexpressing induced differentiation cells. *ASCL2* and *NOTCH1* were not expressed in trophectoderm but were differentially expressed in cell models (Figure 4G). Embryonic implantation sites and non‐implantation sites of the trophectoderm express different genes involved in various cellular processes of embryonic implantation. We analysed the effects of *CDX2* overexpression on genes common to human polar and mural trophectoderm using differential gene analysis and functional annotation, Proliferation activity (*CCNB2* and *CCND1*), ATP synthesis (*NDUFS5*, *NDUFB7* and *UQCR10*), cell migration (*LCP1* and *MMP2*) and other genes were overrepresented in *CDX2* overexpressing cells. Genes involved in hormone stimulation and maternal‐foetal dialogue (*TEAD3* and *ARID3A*) and functional maintenance of mTOR (*PIK3C2A*, *EGFR* and *FOXO4*), an important signalling pathway regulating maternal and foetal nutrient metabolism, were downregulated to varying degrees in both CDX2 overexpressing hTSCs and syncytiotrophoblasts (Figure 4H). These results indicate that *CDX2* is first downregulated in the polar trophectoderm during implantation, which can create conditions for initiating trophoblast lineage differentiation and mother‐to‐foetus communication. The expression of *CDX2* may be important in promoting proliferation and migration of mural trophectoderm.

In summary, our findings suggest that *CDX2* can inhibit the differentiation of human trophoblast stem cells into syncytiotrophoblasts in response to external stimuli, and is among the prominent markers to distinguish polar trophectoderm from mural trophectoderm. Although *CDX2* overexpression impedes syncytialization by delaying hTSCs fusion, it preserves the robust migratory and invasive capabilities of primary syncytiotrophoblast during implantation. This underscores the prominent role of *CDX2* in maintaining the orderly differentiation of early hTSCs.

### 3.5 Early invasion and migration of hTSCs facilitated by *CDX2* overexpression

**FIGURE 5:**
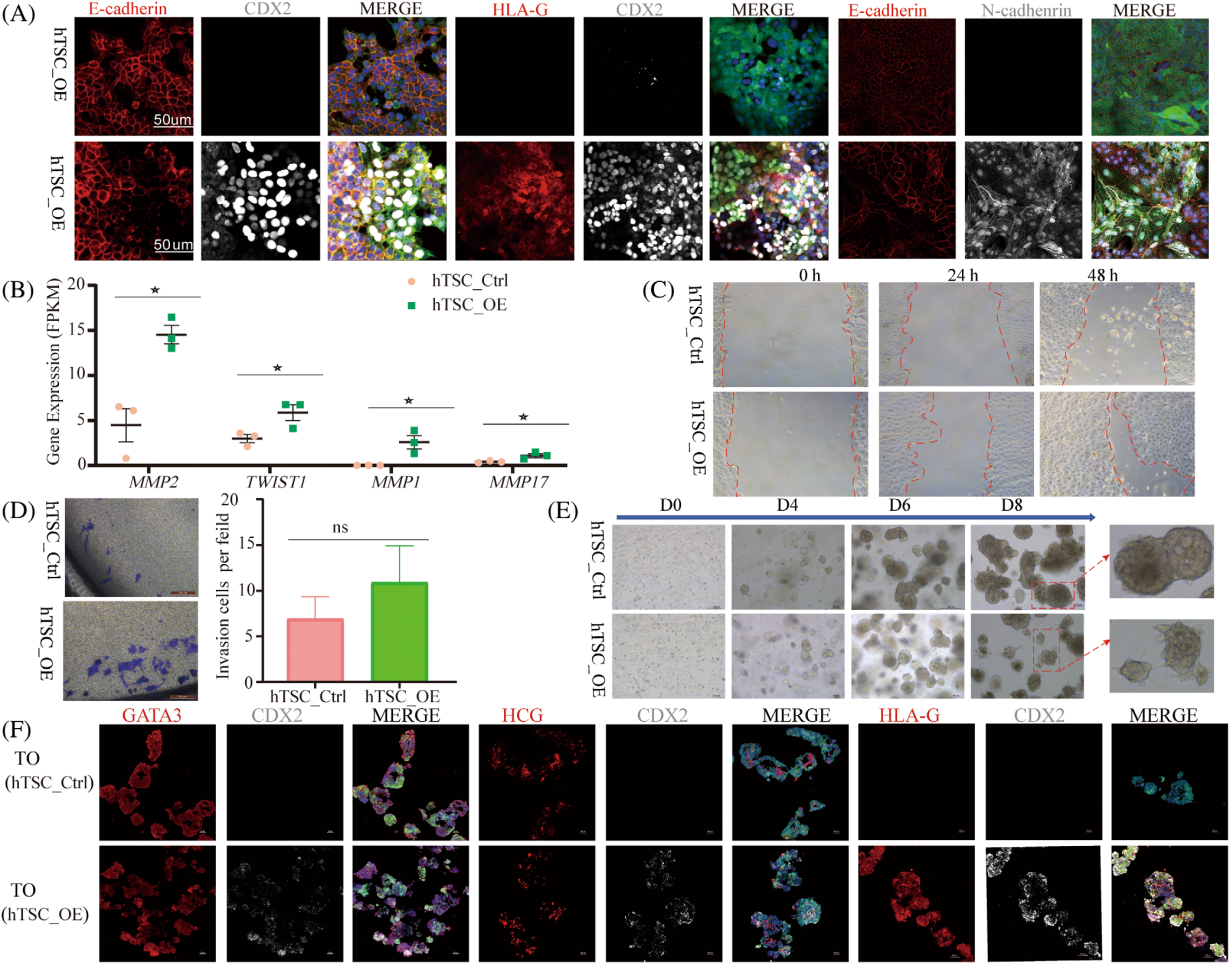
*CDX2* overexpression facilitates the invasion and migration of human trophoblast stem cell (hTSCs). (A) Immunofluorescence staining for epithelial and mesenchymal markers in hTSC_Ctrl and hTSC_OE. Epithelial cell for *E‐cadherin* staining; *HLA‐G*, human leukocyte antigen G, *N‐cadherin*, marker for mesenchymal cells, they were used to detect the effect of *CDX2* on epithelial mesenchymal transition in hTSCs. Scale bars are 50 μm; (B) fragments per kilobase of transcript per million mapped reads (FPKM) chart of marker genes for recognised invasion and migration in hTSC_Ctrl and hTSC_OE, data are presented as mean ± SE (*n* = 3 number of experimental replications), *p ≤ 0.05; (C) scratch assay assessing the migration capability of hTSC_Ctrl and hTSC_OE. Representative images of migrating cells (magnification, ×10). Scale bars are 100 μm; (D) trans‐well assay evaluating the invasion capability of hTSC_Ctrl and hTSC_OE. Representative images of invading cells (magnification, ×10, bar = 100 μm) and statistical analysis of invasive cells. Data are presented as mean ± SE (*n* = 12 number of experimental replications), ns, non‐significant; (E) images of trophoblast organoids for the hTSC_Ctrl and hTSC_OE at different time points. The bottom‐right image is an enlarged view of the area outlined by the red dashed box. (F) Immunofluorescence images of trophoblast organoids in hTSC_Ctrl and hTSC_OE. hTSC for *GATA3* staining; syncytiotrophoblast for *hCG‐β* staining, EVT for *HLA‐G* staining. TO, trophoblast organoid.

It has been reported that *Cdx2* overexpression during the blastocyst stage in mice can hinder trophoblast invasion and migration, thereby resulting in implantation failure.^21^, ^24^ *CDX2* overexpression delayed syncytiotrophoblast differentiation to preserve hTSC characteristics (Figure S2D,E), and the results of differential gene and GO analyses showed that gene expression related to invasive migration was upregulated during *CDX2* overexpression‐related cell differentiation to syncytiotrophoblast (Figure 4E,F). Therefore, we speculated that *CDX2* overexpression could promote the invasive migration function of hTSCs. To further test this hypothesis, we first identified a reduction in the expression of the epithelial marker *CDH1* (*E‐cadherin*) in hTSCs with *CDX2* overexpressing through immunofluorescence. Given the important role of epithelial–mesenchymal transition (EMT) in promoting invasion and migration, we then examined the expression changes of representative mesenchymal genes *HLA‐G* and *CDH2* (*N‐cadherin*) in two hTSC models using the same methods. The results indicate that *CDX2* overexpression promotes EMT in hTSCs (Figure 5A). Additionally, indicator molecules for invasion and migration (*MMP2*, *TWIST1*, *MMP1* and *MMP17*) were detected to be upregulated in *CDX2* overexpressing hTSCs (Figure 5B). Simultaneously, scratch assay and Trans‐well results demonstrate that *CDX2* overexpression enhances the invasion and migration of hTSCs (Figure 5C,D).

Due to the multifactorial interactions between the trophoblast and the endometrium during embryo implantation, this process involves not only the orderly differentiation of the trophoblast but also changes in the spatial structure of the trophoblast.^32^, ^33^ To simulate the temporal–spatial changes in trophoblast development, we referred to the 3D in vitro culture system of trophoblast organoids reported in previous studies.^34^, ^35^ Through optical microscopy, we observed continuous growth of trophoblast organoids with prolonged culture time, and *CDX2* overexpression promoted the formation of villi in trophoblast organoids (Figure 5E). Immunofluorescence results indicated that both the control group and trophoblast organoids constructed by *CDX2* overexpressing cells expressed hTSCs marker *GATA3* and syncytiotrophoblast marker *hCG‐β*. However, *CDX2* overexpression enhanced the expression of the Interstitial marker *HLA‐G* in trophoblast organoids (Figure 5F). This suggests that the constructed trophoblast organoids possess the ability for multipotent differentiation, and there is spatial heterogeneity in *CDX2* expression locations concerning syncytialization.

In summary, whether in 2D or 3D culture, the overexpression of *CDX2* consistently enhances the invasive and migratory properties of early hTSCs. This indicates that the expression level of *CDX2* independently influences the regulation of invasion and migration in early hTSCs. While *CDX2* expression is conserved across mammals, its functionality displays species‐specific variations.

### 3.6 *CDX2* binding and suppression of genes associated with syncytiotrophoblast in hTSCs

**FIGURE 6:**
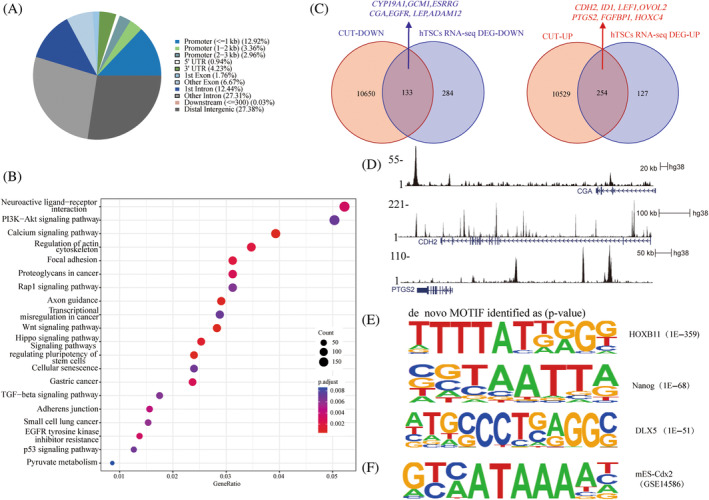
*CDX2* binding and suppression of syncytialization‐related genes in human trophoblast stem cell (hTSCs). (A) Proportion of genomic features overlapping with *CDX2* CUT‐tag peaks in hTSCs; (B) enrichment of KEGG terms for *CDX2* peaks; (C) Venn diagram illustrating the integration between genes bound by *CDX2* (identified by CUT‐tag) and genes with downregulated or upregulated in hTSC_Ctrl and hTSC_OE (based on RNA‐seq analysis). Genes influenced by both *CDX2* binding and these two changes are highlighted with red arrows. Names are indicated in the illustration; (D) RPKM‐normalised *CDX2* binding profiles on selected gene loci determined in panel (C); (E) de novo motif analysis using the HOME software; (F) newly identified motifs similar to *CDX2* that match the *Cdx2* motif preserved in embryonic stem cells of mice via ChIP‐Seq data.

To investigate the target genes regulated by *CDX2* within hTSCs and unravel the molecular pathways governing *CDX2*'s role in trophoblast lineage differentiation, we employed CUT‐tag on the cell model. The analysis unveiled 47,075 *CDX2* binding sites, with nearly 13% residing in the promoter region, 40% in intronic areas and 27% in distant intergenic regions, indicating an enrichment of enhancers within *CDX2* peaks (Figure 6A). The Genomic Region Enrichment Annotation Tool analysis depicted an enrichment of information associated with the regulation of the cytoskeleton, cell adhesion, stem cell pluripotency and the WNT signalling pathway (Figure 6B). We integrated differentially expressed genes related to *CDX2* overexpression in hTSCs with *CDX2*‐binding genes to refine the functional targets. A Venn diagram showed 133 downregulated and 254 upregulated gene sets overlapping between *CDX2*‐binding genes and *CDX2* overexpression differential genes. The downregulated gene set included *GCM1*, *CYP19A1*, *CGA*, *ESRRG* and *EGFR*, and the upregulated gene set included *CDH2*, *ID1*, *LEF1*, *PTGS2* and *FGFBP1* (Figure 6C). We performed Integrative Genomics (IGV) analysis to demonstrate that *CDX2* could bind to *CGA*, *CDH2* and *PTSG2* (Figure 6D). These genes are direct targets of *CDX2*, intimately linked to trophoblast fusion and invasion, although their precise functional roles need further elucidation.

To gain a deeper understanding of the gene regulation driven by *CDX2*, we conducted a de novo motif analysis using HOMER. The identified motifs, including *HOXB13*, *Nanog*, *DLX5* and *TFAP2A*, showed a high degree of similarity to the *CDX2* motif (Figure 6E). Comparison of the de novo motif of *CDX2* with the published *Cdx2* motif confirmed its identity (Figure 6F), indicating potential interactions between these factors and *CDX2*. Some members of the *HOX*, *TFAP2* and *DLX TF* families have been reported as critical regulators of trophoblast fate in both humans and mice. Moreover, *Nanog*, *DLX5*, and *TFAP2A* are known to cooperatively regulate early trophoblast differentiation in vitro. In summary, our findings emphasise the crucial role of *CDX2* as a key regulatory factor in the transcription factor network governing syncytialization.

CUT‐tag analysis showed that *CDX2*‐bound genes were enriched in the WNT signalling pathway, and that *GCM1* and *LEF1* were *CDX2*‐binding targets in hTSCs (Figure 6B,C). The lymphoid enhancer‐binding factor 1 (*LEF1*), a key nuclear factor in the WNT/β‐catenin pathway, binds to β‐catenin regulates the expression of multiple target genes, thereby regulating cell proliferation survival and invasive migration.^36^ Glial cell deletion‐1 (*GCM1*) is a transcription factor that is a master regulator of human syncytiotrophoblast formation and regulates the differentiation of hTSCs into the extravillous trophoblasts by inducing the expression of the master regulator, *ASCL2* and the WNT antagonist, *NOTUM*, whose aberrant expression can lead to impaired trophoblast invasive migration function.^37^ We observed that in *CDX2* overexpressing trophoblast cells, along with the downregulation of *GCM1* expression, those of *ASCL2* and *NOTUM* were downregulated (Figure 4G), consistent with previous reports.^37^ However, *CDX2* overexpression in hTSCs enhanced invasive migration function and upregulated the expression of genes related to the WNT signalling pathway (Figures 3E and 5). The WNT signalling pathway is a complex regulatory network with multiple branches. Therefore, we speculated that *CDX2* could activate the endogenous classical WNT signalling pathway activity in hTSCs and regulate the development and function of hTSCs through additional pathways of the WNT signalling cascade.

To verify this hypothesis, first, we compared the changes in the expression of genes, including *KI67*, *TP63* and *β‐catenin* in hTSCs during the culture of *CDX2* overexpressing hTSCs with or without the addition of exogenous small‐molecule activator of the WNT signalling pathway CHIR99021. The results showed that the *CDX2*‐positive hTSCs expressed the following genes without adding CHIR99021: proliferation marker genes including *KI67*, *TP63* and *β‐catenin* (Figure S3A). However, the number of *CDX2*‐positive hTSCs was reduced under this condition, and few hTSCs showed *β‐catenin* entry into the nucleus around the nucleus. This suggests that although *CDX2* expression in hTSCs depends on exogenous classical WNT signalling pathway activators, it can activate the endogenous WNT pathway to complement its signalling stimulus and thus maintain its stem cell characteristics and functions. Using the same method, we examined the effect of classical WNT signalling pathway activation intensity on the spontaneous differentiation into syncytiotrophoblast, and the results showed that with the absence of exogenous activators, *CDX2* overexpressing hTSCs cells were *hCG‐β* positive. *GCM1* expression was changed from nuclear to cytoplasmic fraction (Figure S3B). Therefore, we hypothesised that *CDX2* could regulate the expression mode of *GCM1* through the classical WNT signalling pathway, thus affecting syncytiotrophoblast formation.

Next, we exposed hTSCs and syncytiotrophoblast differentiation cultures under *CDX2* overexpression to a small‐molecule inhibitor of the WNT pathway at a concentration of 2 μM and evaluated the effects on the proliferation of hTSCs and syncytiotrophoblast differentiation and the position of *GCM1* expression. In *CDX2* overexpressing hTSCs, the addition of the inhibitor at the same concentration as the activator did not change the proliferative activity of the cells (Figure S3B,C). In contrast, the addition of a small‐molecule WNT inhibitor ameliorated the defective fusion of hTSCs caused by *CDX2* during syncytium differentiation, while promoting the shift from nuclear to cytoplasmic expression of GCM1, a phenomenon that was more pronounced in the IWP2‐added group. (Figure S3D).

## 4 DISCUSSION

The influence of trophoblast differentiation on embryo implantation has attracted significant attention. Currently, various trophoblast cell models, including human choriocarcinoma cells and cells derived from the placental lineage, have shed light on the signalling pathways and crucial transcription factors involved in syncytialization, invasion, and migration processes. These findings serve as a basis for refining subsequent studies regarding in vitro culture and differentiation for trophoblast stem cells.^38^ Advancements in biotechnology have further expanded the sources of human trophoblast cells, encompassing derivation from blastocysts and induction from human embryonic stem cells. Consequently, more sophisticated in vitro culture systems have been established.^27^, ^39^, ^40^, ^41^ Additionally, detailed insights into the transcriptome during early trophoblast differentiation and the sublineage classification of trophoblast cells have been provided through single‐cell sequencing of trophoblast cells collected from in vitro cultured embryos.^42^, ^43^, ^44^ While *Cdx2* has been identified as a critical transcriptional activator for trophoblast development in mice and has been implicated in the epigenetic regulation of the IFNT gene during early trophoblast development in cattle, contributing to the modulation of foetal–maternal communication in ruminants, there remains a lack of suitable cell models for investigating the function of *CDX2* in human trophoblast due to its specific spatiotemporal expression characteristics.^45^, ^46^ In this study, we successfully established an in vitro model of expressing *CDX2* hTSCs, enabling us to observe the function of *CDX2* in the development of hTSCs.

In humans, embryo implantation occurs on the sixth day after fertilisation. During this period, the polarised trophectoderm of the blastocyst is primarily composed of two parts: the polar trophectoderm adjacent to the inner cell mass and the mural trophectoderm away from the inner cell mass. These two types of trophectoderm exhibit significant differences in gene expression and functionality.^31^, ^47^ The polar trophectoderm makes initial contact with the uterine endometrium and gradually differentiates into a multinucleated syncytiotrophoblast in response to signals from the uterine endometrium. However, the fate changes of the mural trophectoderm during implantation remain unknown.^48^, ^49^ Previous studies have reported that the expression level of *Cdx2* is one of the determining factors in trophectoderm differentiation and implantation of mice. Through a 3D delayed in vitro culture system of human embryos and sequencing data,^22^ we elucidated the connection between *CDX2* expression changes in polar and mural trophectoderm, as well as lineage differentiation during implantation. *CDX2* is preferentially downregulated in polar trophectoderm during implantation, but *CDX2* expression is maintained in mural trophectoderm, and its downregulation is a prerequisite for the exclusion of molecular signalling from the maternal endometrium to stimulate differentiation into syncytiotrophoblast. The trophoblast is a major member of the constitutive placenta, and its function and fate are closely related to the functional changes of the placenta during the gestation cycle, with significant differences in the heterogeneity of the trophoblast at different times.^3^, ^11^, ^50^ With the onset of pregnancy and the increase in foetal nutritional requirements, the placental delivery of nutrients will change from tissue‐based to blood‐based nutrition, during which adequate trophoblast stem cells are required to differentiate into syncytiotrophoblasts and extravillous trophoblasts in a timely manner, as well as to enhance the trophoblast's invasive migratory capacity to transform the maternal spiral arteries, thereby forming a blood curtain placenta.^4^, ^9^ The trophoblast assumes the responsibility of safeguarding maternal and foetal health in the early and middle stages of pregnancy, while in the latter stages of pregnancy, the placental function deteriorates and the proportion of trophoblast senescence and apoptosis gradually increases.^51^ As *CDX2* serves as one of the markers for hTSCs, our established cell model also confirmed that *CDX2* overexpression had a positive impact on hTSCs proliferation and activity. Furthermore, a multi‐dimensional analysis from both the cellular and genetic levels revealed that, although *CDX2* overexpression delayed hTSCs fusion to inhibit syncytialization, it promoted the expression of genes associated with EMT in the trophoblast and enhanced cell invasion and migration capabilities. Mariko Horii's team also reported that *CDX2* is highly expressed in trophoblast stem cells at 6 weeks of gestation until it disappears again at 20 weeks,^52^ and that these stages are also critical for the transformation of the organised nutritive placenta to the haematopoietic placenta, and that the dynamic expression of *CDX2* at this stage may be for the purpose of increasing the reserve of the trophoblast stem cell pool and promoting the remodelling of the spiral arteries of the uterus, which will in turn maintain pregnancy.

The trophectoderm undergoes early‐stage differentiation into various subtypes of trophoblast cells to sustain the necessary nutritional requirements for embryonic growth and development, forming the earliest functional placenta. Despite the initial insights into the differentiation processes of the early trophoblast during embryo implantation and its interaction with the uterine microenvironment,^12^, ^50^, ^53^, ^54^ many key molecules involved in embryo implantation and the molecular pathways regulating trophoblast lineage differentiation remain largely unknown. The trophoblast undergoes drastic changes during implantation, and trophoblast organoids serve as ideal models for simulating trophoblast development. They provide a comprehensive representation of the complex structure, biological and functional characteristics of different trophoblast subpopulations, thus bridging the gap between in vitro and in vivo models.^34^, ^35^ In our study, we successfully constructed *CDX2*‐overexpressing organoids using a 3D trophoblast organoid culture medium. Through the analysis of these organoids, we further elucidated that *CDX2* overexpression promotes hTSCs EMT. Combining CUT‐tag, we found that *CDX2* can inhibit premature syncytiotrophoblast differentiation by directly regulating key genes in syncytiotrophoblast formation, such as *CGA*, *GCM1*, the EMT‐related gene *CDH2*, and invasion‐migration‐related gene *PTGS2*. The process of embryo implantation is a complex interplay of various cells and molecules, finely regulated. *CDX2* dynamically expresses in the trophectoderm of pre‐ and post‐implantation blastocysts. Exploring molecular indicators influencing *CDX2* expression can provide a valuable reference for assessing embryo implantation. Although our results have preliminarily indicated that the expression of *CDX2* in hTSCs is closely related to the activation of the classical WNT signalling pathway and that *CDX2* can exert a pro‐proliferative effect by amplifying WNT signals in hTSCs, and that *CDX2* can intervene in the formation of the syncytiotrophoblast by regulating the localisation of *GCM1* in the cells via the WNT signalling pathway, However, the specific molecular mechanism remains to be further explored.

Overall, we have elucidated the correlation between *CDX2* and trophoblast lineage differentiation and the effects of *CDX2* overexpression on syncytiotrophoblast formation, hTSCs proliferation, and invasive migration by different research methods, revealing the role of *CDX2* in the human trophoblast lineage differentiation during the implantation period, which may provide a new perspective for the etiological screening of patients with implantation failure in the clinic. However, due to the lack of a culture system to maintain endogenous *CDX2* expression in cells and the large functional differences of *CDX2* in different species, investigating its function at the in vivo and human embryonic levels poses challenges. Therefore, in the future, it is necessary to further improve the system of induced differentiation of embryonic stem cells and to search for indicators that can be used to targetively assess the changes of *CDX2* in vivo during the implantation period. In addition, *CDX2* expression was detected in amniocytes of Day 9 inner cell mass origin, and the formation of the amniotic cavity is crucial for establishing the bilaminar embryonic disc structure in primates.^55^, ^56^, ^57^ This may indicate the multiple functions of *CDX2* in embryonic development, and it would be interesting to further investigate the role of *CDX2* in the development and morphological remodelling of the primate embryo by using the class of embryos in the future.

## AUTHOR CONTRIBUTIONS

ZW and TL initiated the project and supervised the entire project. ZW and LR designed the experiments and wrote the manuscript. TL supervised ZA and BN to perform data analysis. YW, YY, XL, YB, XZ and XL performed stem cell isolation and culture, transfection, organoid construction. LX, HZ, YK, performed human embryo culture. CF, GS, TC and JY contributed to sequencing data analysis and manuscript preparation.

## CONFLICT OF INTEREST STATEMENT

The authors declare no competing interests.

## Supporting information


**Data S1.** Supporting Information.

## Data Availability

The clean sequence data reported in this paper have been deposited in the Genome Sequence Archive (Genomics, Proteomics & Bioinformatics 2021) in National Genomics Data Center (Nucleic Acids Res 2022), China National Center for Bioinformation/Beijing Institute of Genomics, Chinese Academy of Sciences (GSA‐Human: HRA010392) that are publicly accessible at https://ngdc.cncb.ac.cn/gsa-human. Human embryo scRNA‐seq data have been deposited in the Gene Expression Omnibus (GEO) under accession number GSE136447 (scRNA‐seq data).^30^
