# Supplementary material for: The impact of dynamic caudal type homeobox 2 expression on the differentiation of human trophoblast lineage during implantation
Source: Cell Prolif. 2024 Aug 19;57(12):e13729. doi: 10.1111/cpr.13729 (PMC11628739; doi:10.1111/cpr.13729)
Supplement: Supplementary file 1 — Data S1. Supporting Information. [file CPR-57-e13729-s001.pdf]

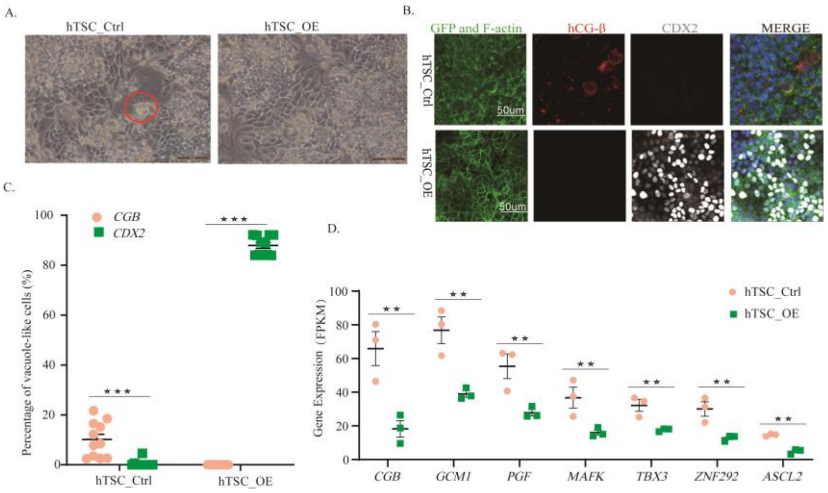

Supplement Figure 1. Impact of *CDX2* overexpression on spontaneous differentiation of hTSC

A. Bright-field images on the 4th day under hTSC culture conditions for hTSC_Ctrl and hTSC_OE. Red circles are vacuolar structures formed by hTSC spontaneous differentiation, Scale bars are 200 μm;

B. Immunofluorescence images of syncytiotrophoblast markers on the 4th day after hTSC culture for hTSC_Ctrl and hTSC_OE, Scale bars are 50 μm;

C. Expression of syncytiotrophoblast marker genes in vacuole-like cells. Analysis of changes in the expression of *CGB* and *CDX2* in hTSC_Ctrl and hTSC_OE, and preliminary assessment of the correlation between CDX2 and syncytiotrophoblast. Data are presented as mean ± SE (n = 11 number of experimental replicates); ***$p \leq 0.001$.

D. Transcriptome sequencing analysis of changes in the expression of classical markers for syncytiotrophoblast in stem cells between hTSC_Ctrl and hTSC_OE. Data are presented as mean ± SE (n = 3 number of experimental replications)， **$p \leq 0.01$.

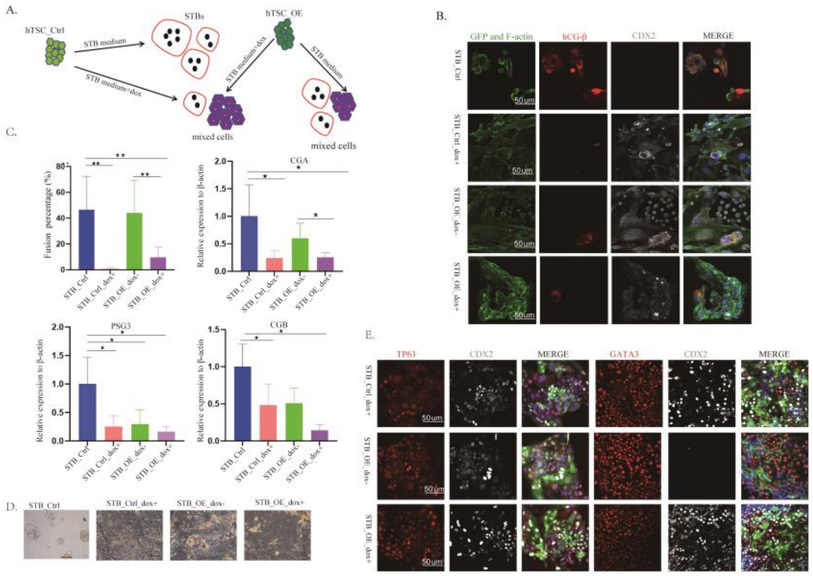

Supplemented Figure 2. Delayed syncytialization caused by *CDX2* overexpression

A. Cell models and grouping under directed differentiation;

B. Immunofluorescence illustrating the proportion of syncytialization and *CDX2* expression changes after six days of directed differentiation under the conditions outlined in Panel A, Scale bars are 50 μm;

C. Statistical analysis of syncytialization. Syncytia are defined as cells with at least three nuclei. The number of nuclei and syncytia stained with DAPI is calculated. The syncytialization can be quantified using the formula: $[(N-S)/T] \times 100$, where N is the number of syncytial nuclei, S is the number of syncytia, and T is the total number of counted nuclei. Data are presented as mean ± SE (n = 5 number of experimental replications), $**p \leq 0.01$. q-PCR analysis of the mRNA expression of syncytiotrophoblast markers *CGA*, *PSG3*, and *CGB* under different experiment conditions. Data are presented as mean ± SE (n = 3 number of experimental replications), $*p \leq 0.05$;

D. Bright-field images of cells cultured under different experiment conditions for six days. Scale bars are 100 μm;

E. Immunofluorescence to identify stem cell characteristics of D-figure cells. *TP63,GATA3* and *CDX2* were used for hTSC characterization , Scale bars are 50 μm.

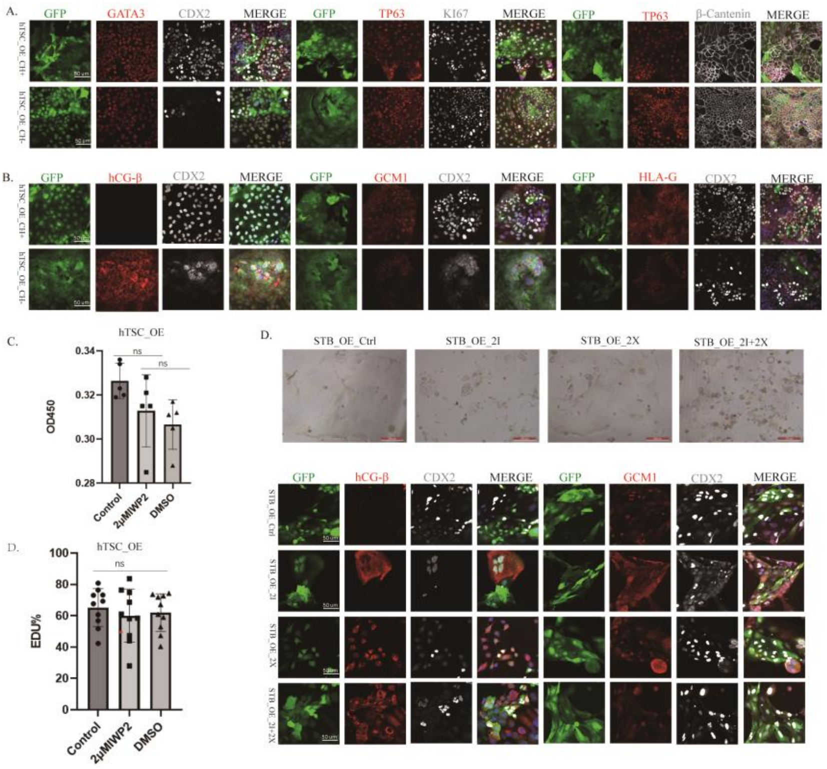

Supplemented Figure 3. *CDX2* affects syncytial trophectoderm differentiation by regulating the spatial expression position of *GCM1* through the classical WNT signalling pathway

A. Immunofluorescence was used to detect changes in pluripotency marker gene expression in hTSC_OE cells after removal of exogenous classical WNT small-molecule activators, and the correlation between *CDX2* and activation of the classical WNT signalling pathway. *GATA3, TP63* and *CDX2* were used to detect the effect of activation of the exogenous classical WNT signalling pathway on the maintenance of stem cell identity in hTSC_OE cells, and *β- cantenin* was used to detect the role of

CDX2 in classical WNT activation. hTSC_OE: *CDX2* overexpression in trophoblast stem cells, Scale bars are 50 μm;

B. Immunofluorescence demonstration of the effect of removal of exogenous classical WNT small molecule activators on the spontaneous differentiation of hTSC_OE into syncytiotrophoblasts and interstitialisation, and the correlation between activation of the classical WNT signalling pathway and changes in the expression of *GCM1*. *hCG-β* and *GCM1* were used to detect the formation of syncytial trophoblasts by spontaneous differentiation, and *HLA-G* was used to detect interstitialisation. scale bars are 50 μm.

C. CCK8 assay of the effect of the addition of IWP2, a small molecule inhibitor of classical WNT signalling, on the proliferative capacity of hTSC_OE, Control is the addition of tetracycline-induced *CDX2* overexpression of trophoblast stem cells, DMSO is the solvent for IWP2, and the DMSO group in order to reduce experimental errors caused by the solvent. Data are presented as mean ± SE (n = 5 number of replicates), ns：non-significant;

D. Plot of statistical analysis of EDU labelling after exogenous addition of the classical WNT signalling small molecule inhibitor IWP2 to hTSC_OE, Data are presented as mean ± SE (n = 10 number of replicates), ns: non-significant;

E. Immunofluorescence images of GCM1 spatial expression location and syncytiotrophoblast formation during hTSC_OE-induced differentiation after inhibition of the classical WNT signaling pathway. STB_OE_2I :Differentiation medium of overexpressing CDX2 trophoblast stem cells supplemented with 2 μM IWP2, 2X: Differentiation medium of overexpressing CDX2 trophoblast stem cells

supplemented with 2 μM XAV939, 2I+2X: Differentiation medium of overexpressing CDX2 trophoblast stem cells supplemented with 2 μM IWP2 + 2 μM XAV939, STB: syncytiotrophoblast, cale bars are 50 μm.
